# Cannabis consumption is associated with lower COVID-19 severity among hospitalized patients: a retrospective cohort analysis

**DOI:** 10.1186/s42238-022-00152-x

**Published:** 2022-08-05

**Authors:** Carolyn M. Shover, Peter Yan, Nicholas J. Jackson, Russell G. Buhr, Jennifer A. Fulcher, Donald P. Tashkin, Igor Barjaktarevic

**Affiliations:** 1 Division of Pulmonary & Critical Care Medicine, David Geffen School of Medicine at the University of California, Los Angeles, Los Angeles, CA USA; 2 Offsite Medical Care, Intensive Care Telemedicine, Santa Rosa, CA USA; 3 David Geffen School of Medicine at the University of California, Los Angeles, Los Angeles, CA USA; 4 Department of Medicine Statistics Core, David Geffen School of Medicine at the University of California, Los Angeles, Los Angeles, CA USA; 5 Center for the Study of Healthcare Innovation, Implementation, and Policy, Health Services Research & Development, Greater Los Angeles Veterans Affairs Healthcare System, Los Angeles, CA USA; 6 Division of Infectious Diseases, David Geffen School of Medicine at the University of California, Los Angeles, Los Angeles, CA USA

**Keywords:** COVID-19, Cannabis, Outcomes, Respiratory failure, ARDS

## Abstract

**Background:**

While cannabis is known to have immunomodulatory properties, the clinical consequences of its use on outcomes in COVID-19 have not been extensively evaluated. We aimed to assess whether cannabis users hospitalized for COVID-19 had improved outcomes compared to non-users.

**Methods:**

We conducted a retrospective analysis of 1831 patients admitted to two medical centers in Southern California with a diagnosis of COVID-19. We evaluated outcomes including NIH COVID-19 Severity Score, need for supplemental oxygen, ICU (intensive care unit) admission, mechanical ventilation, length of hospitalization, and in-hospital death for cannabis users and non-users. Cannabis use was reported in the patient’s social history. Propensity matching was used to account for differences in age, body-mass index, sex, race, tobacco smoking history, and comorbidities known to be risk factors for COVID-19 mortality between cannabis users and non-users.

**Results:**

Of 1831 patients admitted with COVID-19, 69 patients reported active cannabis use (4% of the cohort). Active users were younger (44 years vs. 62 years, *p* < 0.001), less often diabetic (23.2% vs 37.2%, *p* < 0.021), and more frequently active tobacco smokers (20.3% vs. 4.1%, *p* < 0.001) compared to non-users. Notably, active users had lower levels of inflammatory markers upon admission than non-users—CRP (C-reactive protein) (3.7 mg/L vs 7.6 mg/L, *p* < 0.001), ferritin (282 μg/L vs 622 μg/L, *p* < 0.001), D-dimer (468 ng/mL vs 1140 ng/mL, *p* = 0.017), and procalcitonin (0.10 ng/mL vs 0.15 ng/mL, *p* = 0.001). Based on univariate analysis, cannabis users had significantly better outcomes compared to non-users as reflected in lower NIH scores (5.1 vs 6.0, *p* < 0.001), shorter hospitalization (4 days vs 6 days, *p* < 0.001), lower ICU admission rates (12% vs 31%, *p* < 0.001), and less need for mechanical ventilation (6% vs 17%, *p* = 0.027). Using propensity matching, differences in overall survival were not statistically significant between cannabis users and non-users, nevertheless ICU admission was 12 percentage points lower (*p* = 0.018) and intubation rates were 6 percentage points lower (*p* = 0.017) in cannabis users.

**Conclusions:**

This retrospective cohort study suggests that active cannabis users hospitalized with COVID-19 had better clinical outcomes compared with non-users, including decreased need for ICU admission or mechanical ventilation. However, our results need to be interpreted with caution given the limitations of a retrospective analysis. Prospective and observational studies will better elucidate the effects cannabis use in COVID-19 patients.

**Supplementary Information:**

The online version contains supplementary material available at 10.1186/s42238-022-00152-x.

## Background

The COVID-19 (Coronavirus Disease 2019) Pandemic caused by SARS-CoV2 (severe acute respiratory syndrome coronavirus 2) has upended the global community, infecting over 300 million people and claiming over 5 million lives in its wake (Coronavirus Disease (COVID-19) Situation Reports n.d.). In case surveillance data from the Centers for Disease Control and Prevention (CDC) during January to May of 2020, 14% of patients were hospitalized, 2% required ICU (intensive care unit) admission and 5% died (Gold et al. 2021). In the USA case surveillance reports from 2020, during the time our data was collected, found that 14% of patients required hospitalization, 2% required ICU admission and 5% of all patients died (Gold et al. 2021). While several studies have reported the detrimental effects of tobacco smoking on outcomes in patients with COVID-19(Saadatian-Elahi et al. 2021; Patanavanich and Glantz 2020), little is known about these outcomes in people who use cannabis products. Cannabis has long been known to have immunomodulatory effects and thus may not have the same negative effects as tobacco in patients with COVID-19 (Colmenero et al. 2021).

The immunomodulatory effects of cannabis may be especially important to understand in hospitalized patients with COVID-19, where progression to more severe forms of COVID may be due to manifestations of hyperinflammatory states (Ioannidis 2020). Patients with severe COVID-19 have elevated clinical inflammatory markers (CRP, ferritin, procalcitonin) and increased serum cytokine and chemokine levels (IL-1B, IL-8, sTNFR) (Han and Mallampalli 2015; Pink et al. 2021). Thus, one general hypothesis is that dampening the immune system after infection with COVID may be a plausible strategy for mitigating severe disease progression (Onaivi and Sharma 2020). Indeed, current guidelines from the CDC for treating patients with severe COVID-19 include immunosuppressive medications such as dexamethasone, tocilizumab and barcitinib (Hospitalized Adults: Therapeutic Management n.d.). Furthermore, liver transplant patients on immunosuppressants who contracted COVID were found to have lower mortality rates compared to the matched general population in a nationwide prospective study conducted in Spain (Guillon et al. 2020).

Phytocannabinoids (CBD) found in cannabis are capable of binding to endogenous cannabinoid receptors CB_1_ and CB_2._ These receptors are commonly expressed in neural tissue and immune cells, respectively. Notably, CB_2_ receptors have been proposed as a therapeutic target for multiple sclerosis when it was shown that selective CB_2_ agonism could ameliorate symptoms of experimental autoimmune encephalomyelitis in mice through suppression and apoptosis of encephalitogenic T cells (Paland et al. 2021). More relevantly, treatment of SEB-induced (staphylococcal enterotoxin B) acute respiratory distress syndrome (ARDS) in mice with tetrahydrocannabinol treatment (a partial CB_1/2_ agonist) led to 100% survival, decreased lung inflammation, and suppressed cytokine storm (Maresz et al. 2007). Additionally, several strains of *C. sativa* have also been shown to mitigate SARS coronaviruses by downregulating ACE2 receptors on 3D human tissue models of oral, airway, and intestinal tissues (Mohammed et al. 2020). In a recent study, cannabinoid acids were notably found to prevent entry of SARS-CoV-2 into cells, proposing another mechanism by which cannabis may play an important role in patients with COVID-19 infection (van Breemen et al. 2022).

While some authors recommend caution in encouraging cannabis use for prevention or treatment of COVID-19, as has been eschewed in the media, there has been growing interest in this area (Borgonhi et al. 2021). Recently, a pharmaceutical company has announced an early trial evaluating a cannabidiol treatment in severe and critical COVID-19 infections (Biotechs n.d.).

To further understand how cannabis use affects COVID-19 outcomes, we performed a retrospective analysis of patients admitted to two California hospitals with a diagnosis of COVID-19 and compared cannabis users to non-users. Important for our population, recent prevalence data demonstrated a higher proportion of cannabis users in California compared to the national average, especially in those over age 65 (Recent Trends in Marijuana Use In Los Angeles County 2018). We hypothesize that chronic cannabis use may have positive effects on COVID-19 outcomes in hospitalized patients due to its immunomodulatory and hypothetical anti-viral effects. We hypothesized that those who had chronically used cannabis products and were hospitalized with COVID-19 would have more limited progression to severe disease and reduced mortality compared to non-users.

## Methods

### Study design and inclusion criteria

We performed a retrospective analysis using a database of 1831 patients aged 18 years or older admitted to Ronald Reagan UCLA Medical Center or UCLA Santa Monica Medical Center between February 12, 2020 and February 27, 2021 with a diagnosis of COVID-19 as defined by a positive PCR test at the time of admission. Institutional Review Board (IRB) approval was obtained prior to data collection (IRB 20-000473) with informed consent waived by the IRB given the retrospective nature of the study utilizing existing clinical data. Cannabis use was assessed by patient self-report as part of the patient’s social history obtained at the time of admission. The electronic health records of documented cannabis users were manually abstracted to confirm active use, defined as any use of inhaled (both vaporized and combusted) or edible cannabis within 1 month prior to admission. Patients with missing social history regarding cannabis use were presumed to be non-users. Data was abstracted and stored in a REDCap database (Vanderbilt University, Nashville, TN) (Harris et al. 2009; Harris et al. 2019). UCLA is a large academic center and all patients have been offered up to date medical management over the duration of study, per the recommendations from the CDC (Centers for Disease Control and Prevention) and World Health Organization (WHO).

### Outcome measures and covariate definitions

The primary outcome was NIH COVID-19 severity score, a scale which refers to the eight-category ordinal scale used by the Adaptive COVID-19 Treatment Trial (ACTT) which classifies disease severity from 1 (not hospitalized, no limitations) to 8 (death) (Beigel et al. 2020). Secondary outcomes included need for supplemental oxygen, ICU admission, mechanical ventilation (including duration thereof), length of hospitalization and in-hospital death. We collated patient characteristics by abstracting the electronic health record, including age, body mass index (BMI), and patient-reported sex, race, and tobacco smoking status. We also tabulated comorbidities including diabetes mellitus, cardiac disease, chronic kidney disease, chronic lung disease, and liver disease reported at time of admission, determined using ICD-10 codes. We collected both the admission lab values (first recorded result that was drawn within 48 h of admission) and the highest value that occurred during the patient’s hospital course. We also tallied certain pharmacotherapies commonly used for admitted COVID-19 patients, including remdesivir, systemic corticosteroids, and antibiotics, including use at any point during the patient’s hospital course.

### Statistical analysis

Bivariate comparisons between self-reported active cannabis users and non-users (e.g., former and never users) were assessed using Welch’s *t* tests (or Wilcoxon rank sum) for continuous variables and Fisher’s exact tests for categorical variables. Because of the observational nature of the study and potential bias due to self-selection into the cannabis user groups, we utilized inverse-probability-weighted regression adjustment (IPW-RA) based on the aforementioned covariates (demographics, tobacco smoking status, comorbidities and COVID-specific treatment) as a means to estimate the effect of cannabis use on the study outcomes. The covariates were used in both the creation of the propensity weight and in the regression adjustment portion of the analysis. Both age and BMI were modeled using quadratic terms in order to capture potential non-linear associations with the outcomes and propensity for cannabis use. Certain outcomes (e.g., duration of mechanical ventilation and length of stay (LOS)) were log-transformed in these models. Because unknown tobacco smoking status was collinear with cannabis use (i.e., tobacco smoking status was known for all cannabis users), the IPW-RA models were implemented excluding the 72 non-users with unknown tobacco smoking status. For some outcomes (death, ICU admission, and invasive ventilation), these IPW-RA models failed to converge and a 1-to-1 Propensity Score Matching (PSM) method with caliper distance restricted to within 0.20 was implemented instead. The standardized mean difference between current marijuana users and non-users on the covariates used in the IPW-RA and PSM models were additionally examined (Supplemental Table 2). For the IPW-RA analyses, a post-hoc test for covariate balance was assessed using an omnibus test for over identification. All analyses were conducted in Stata SE version 16.1 (StataCorp, LP, College Station, TX).

## Results

### Demographics and comorbidities

A total of 1831 patients with COVID-19 were included in this study. Of those, 69 (4% of the overall cohort) were active users, defined as use within the past 1 month (Moore et al. 2005). Previous or former users included individuals who had history of cannabis use, but not within 1 month of hospitalization. Demographics, tobacco use, and comorbidities are described in Table 1. Notably, subjects who consumed cannabis were significantly younger than non-users (44 years vs. 62 years, *p* < 0.001). Active users also had lower rates of diabetes mellitus (23.2% vs. 37.2%, *p* < 0.021). There were no significant differences among the other comorbid conditions. Subjects that consumed cannabis also had nearly 5 times higher prevalence of current tobacco smoking (*p* < 0.001).

**Table 1:** Baseline patient characteristics comparing subjects who did and did not actively consume cannabis products

Demographics	Active cannabis users	Non-users or previous cannabis users	***P*** value
	(***n*** = 69)	(***n*** = 1762)	
^a^Age (years), *mean ± SD*	44 ± 19	62 ± 19	< .001
^b^Male sex, %	62	55	0.219
^a^BMI (kg/m^2^), mean ± SD	28.2 ± 7.9	28.8 ± 7.4	0.554
^**b**^**Race, column %**			
White	48%	31%	0.005
Black	15%	9%	0.136
Asian	4%	8%	0.362
Latinx	28%	42%	0.017
Unknown/mixed race	6%	10%	0.401
^**b**^**Tobacco use, column %**			
Never	55%	66%	0.053
Former use	24.6%	25.4%	1.000
Current use	20%	4%	< .001
Unknown use	0^c^%	4%	0.110
^**b**^**Comorbid conditions, column %**			
Diabetes mellitus	23%	37%	0.021
Cardiac disease	16%	24%	0.148
Chronic kidney disease	17%	27%	0.094
Chronic pulmonary disease	26%	26%	1.000
Chronic liver disease	3%	5%	0.581

Active cannabis use defined as cannabis consumption within 1 month of hospitalization. Any cannabis use thereafter are previous users. Non-users are patients with no history of cannabis use

*BMI* body mass index

^a^Welch’s *t* test

^b^Fisher’s exact test

^c^ % of each level of variable within that particular group, not between groups

### Hospitalization outcomes

Based on univariate analysis in Table 2, cannabis users had significantly better outcomes compared to non-users as reflected in lower NIH scores (5.1 vs. 6.0, *p* < 0.001); an NIH score of 5 includes patients requiring supplemental oxygen while a score of 6 differentiates those that require either non-invasive ventilation or high flow oxygen devices. Cannabis users also had shorter hospitalizations (4 days vs. 6 days, *p* < 0.001) and appreciably lower ICU admission rates (12% vs. 31%, *p* < 0.001). In addition, cannabis users had less need for mechanical ventilation (6% vs. 17%, *p* = 0.027) compared to non-users. Using propensity matching, differences in overall survival were not statistically significant between cannabis users and non-users, nevertheless ICU admission was 12 percentage points lower (*p* = 0.018) and intubation rates were 6 percentage points lower (*p* = 0.017) in cannabis users after adjusting for covariates including age, sex, BMI, tobacco smoking history, and COVID-specific treatment (Table 3). No differences were detected in oxygen supplementation between cannabis users and non-users after covariates were accounted for.

**Table 2:** Propensity-matched outcomes comparing active cannabis users and non-users

Outcome	Active cannabis users	Cannabis non-users	Propensity based user effect	***P*** value
			**β** (**95% CI)**	
^1^NIH Score, *mean (SD)*	5.1 (1.2)	6.0 (1.1)	− 0.49 (− 0.69, − 0.29)	< .001
			***e***^***β***^ **(95% CI)**	
^1,a^Intubation duration (days), *median [IQR]*	10.0 [4.0, 20.0]	7.0 [2.0, 13.0]	NC	NC
^1,a^Length of stay (days), *median [IQR]*	4.0 [2.0, 8.0]	6.0 [3.0, 12.0]	0.86 (0.76, 0.98)	< .001
			**OR (95% CI)**	
^2^In-hospital mortality, *%*	4.3%	11.3%	0.98 (0.93, 1.04)	0.565
^1^Oxygen therapy, *%*	50.7%	84.0%	0.88 (0.70, 1.11)	0.270
^2^ICU Admission, *%*	11.6%	30.8%	0.88 (0.80, 0.98)	0.018
^2^Mechanical ventilation, *%*	5.8%	16.6%	0.94 (0.89, 0.99)	0.017

*NIH* National Institutes of Health, *IQR* interquartile range, *NC* non-convergence, *OR* odds ratio

^1^Inverse-Probability-Weighted Regression Adjustment

^2^Propensity-score matching

^a^Log-transformed for modeling

**Table 3:** Care patterns: use of pharmacotherapeutic agent during hospitalization

^**a**^Treatment, %	All	Active users	Non-users	***P*** value
Systemic Steroids	58.2	39.1	59.0	0.001
Remdesivir	54.4	26.1	55.5	< .001
Antibiotics	66.0	49.3	66.6	0.004

^a^Fisher’s exact test

### Inflammatory biomarkers

We evaluated the admission lab values (drawn within 48 h of admission) and the highest value during the patient’s hospital course (Table 4). Notably, cannabis users were more likely to have lower inflammatory markers levels on admission, such as CRP (3.7 mg/L vs. 7.6 mg/L, *p* < 0.001), Ferritin (282 μg/L vs. 622 μg/L, *p* < 0.001), and D-dimer (468 ng/mL vs. 1140 ng/mL, *p* = 0.017) compared to non-users. This effect was sustained during their hospital course, with cannabis users continuing to have lower inflammatory markers compared to non-users. Cannabis users were more likely to have higher lymphocyte counts on admission and during their hospital course. Peak neutrophil counts were also lower in cannabis users, while admission neutrophil values were the same. No statistical difference was detected in total white blood cell (WBC) count between cannabis users and non-users.

**Table 4:** Comparison of laboratory values at admission and peak value during the hospital stay comparing subjects who did and did not use cannabis

^**a**^Lab values, median [IQR]	Active cannabis users	Non-users	***P*** value
White blood cell total count (10^9^/L)			
Admission	6.54 [4.85, 9.44]	6.34 [4.36, 9.37]	0.691
Peak value	9.7 [6.8, 13.6]	10.8 [7.6, 15.3]	0.069
Absolute lymphocyte count (10^9^/L)			
Admission	1.41 [0.73, 2.00]	0.86 [0.57, 1.31]	< .001
Peak value	2.01 [1.37, 2.70]	1.67 [1.17, 2.25]	0.020
Absolute neutrophil count (10^9^/L)			
Admission	4.69 [2.87, 6.63]	4.56 [2.93, 7.32]	0.556
Peak value	6.72 [4.74, 10.21]	7.98 [5.37, 11.99]	0.029
Interleukin 6 (IL-6), pg/mL			
Admission	2.5 [2.0, 6.9]	6.7 [2.0, 17.7]	0.108
Peak value	2.5 [2.0, 15.7]	7.5 [2.5, 21.6]	0.120
C-reactive protein (CRP), mg/dL			
Admission	3.7 [0.6, 6.0]	7.6 [3.0, 13.3]	< .001
Peak value	3.4 [0.8, 8.3]	9.6 [4.2, 16.0]	< .001
Serum ferritin, ng/mL			
Admission	282 [156, 519]	622 [293, 1,262]	0.001
Peak value	287 [125, 645]	778 [347, 1,690]	< .001
D-dimer, ng/mL			
Admission	468 [401, 1,549]	1,140 [671, 2,073]	0.017
Peak value	521 [399, 1,896]	1,628 [947, 4,351]	0.001
Serum procalcitonin, ng/mL			
Admission	0.10 [0.10, 0.12]	0.15 [0.10, 0.39]	0.001
Peak value	0.10 [0.10, 0.20]	0.19 [0.10, 0.64]	0.006

*IQR* interquartile range

^a^Wilcoxon rank sum test

### Pharmacotherapies

**Fig. 1:**
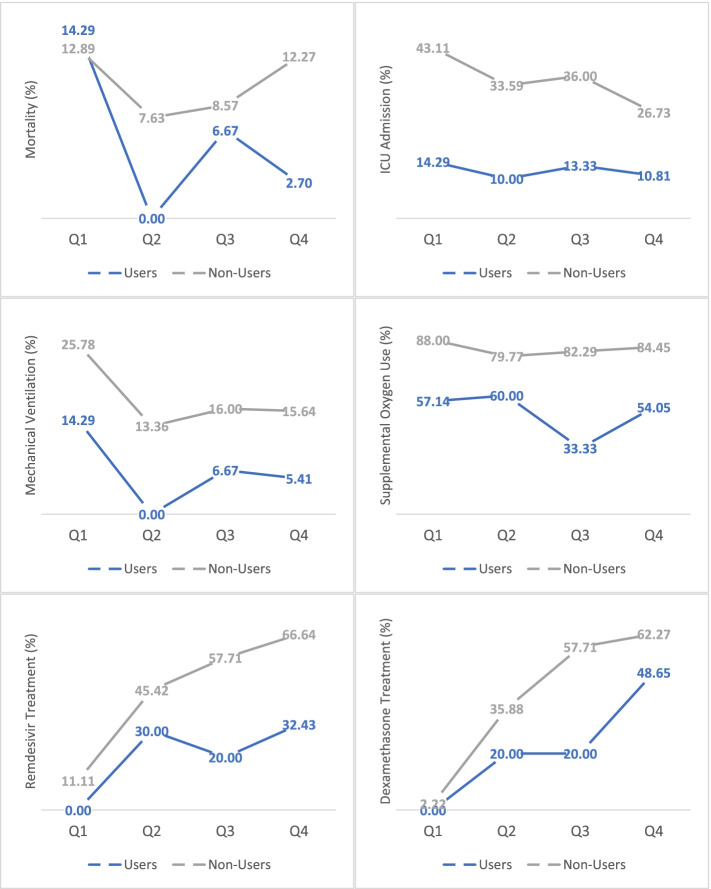
Outcomes and treatment in COVID patients stratified by admission date. Outcomes varied throughout the pandemic due to COVID surges. Cannabis users consistently had less severe disease course. Percentages displayed are the proportion of patients with admission dates within each quarter (Q). Q1: February 2020–May 2020. Q2: June 2020–August 2020. Q3: September 2020–November 2020. Q4: December 2020–February 2021

Given changing recommendations during the time of this patient sample, we also compared the pharmacologic management between groups. We found that non-users were more likely to receive systemic steroids (59.0% vs 39.1%, *p* = 0.001), remdesivir (55.5% vs 26.1%, *p* < 0.001) and antibiotics (66.6% vs 49.3%, *p* < 0.004) compared with users. Thus, cannabis users received less COVID-19 adjunctive therapies but had better outcomes compared to non-users. These findings, along with the aforementioned outcomes investigated in this study, were consistent at all points throughout the pandemic as shown in Fig. 1.

## Discussion

In our study of hospitalized patients with COVID-19 at two Los Angeles area hospitals, we found that cannabis use was associated with decreased disease severity as assessed by NIH severity score and was associated with improved clinical outcomes in COVID-19. Consistent with known trends, active cannabis users were overall younger than non-users. However, when adjusting for age these outcomes remained consistent. Even more, when adjusting for comorbid conditions, demographics and smoking history we found that cannabis users still had less severe disease progression compared to non-users. This remained true despite less utilization of adjunct therapies like remdesivir and corticosteroids. Consistent with our understanding of how cannabis may play a role as an immunomodulator, non-cannabis users were found to have greater elevations in inflammatory biomarkers at the time of admission and during their hospital course. Thus, based on our current understanding of cannabis’ immunomodulatory effects, the link between cannabis usage and better COVID outcomes is sensible.

In the USA in 2020, an estimated 17.9% of the population (49.6 million people) used cannabis during the past year (2020 National Survey of Drug Use and Health (NSDUH) Releases | CBHSQ Data n.d.). Given the magnitude of COVID-19 and the prevalence of cannabis use in the USA, it is important to evaluate how active cannabis usage may affect clinical outcomes in COVID-19 patients. According to a 2019 national survey, past-month cannabis usage in adults has significantly increased over the years from 7.2% in 2016 to 10.2% (Dai and Richter 2019). Important with respect to COVID-19, is a trend of increasing cannabis use in older adults. Han et al conducted a national survey including close to 15,000 respondents and found the prevalence of past-year cannabis use among adults 65 years and older increased from 2.4 to 4.2% from 2015 to 2018, a 75% relative increase (Russell et al. 2018). Given our current knowledge of COVID-19 disproportionately hospitalizing older populations, understanding the effects of cannabis use in COVID patients is increasingly relevant.

In our population of cannabis users, there was a paucity of data regarding route of administration. This is important, as the route of cannabis use can influence health outcomes. While cannabis smoking is associated with respiratory issues including bronchitis and arguably impaired lung function (Tashkin and Roth 2019), some research suggests that vaporized and edible cannabis may offer the potential for reduced health risks, although this has also been questioned (Chadi et al. 2020). Moreover, cannabis vaping has been associated with over 2800 of cases of Electronic or Vaping Associated Acute Lung Injury (EVALI) (The Lancet Respiratory Medicine 2020). The varied pharmacokinetics of cannabis depends on route of administration. Bioavailability of inhaled cannabinoid products is approximately 30% while ingesting cannabis results in 4–12% bioavailability (McGilveray 2005). Peak serum concentration of cannabinoids is also notably higher and achieved more quickly when cannabinoids are inhaled as opposed to ingested. Despite the differences in pharmacokinetics between these two main routes of administration, systematic reviews have shown that there is high user variability within cohorts that all purportedly took in cannabinoids through the same route of administration. Given the diverse ways in which cannabis can be introduced into the body, our grouping of inhaled and ingested cannabis should introduce little variability to an already highly variable cohort of cannabis users. Pooling all cannabis users, regardless of administration method, gives our study more power in analysis while minimizing the risk of overfitted data.

Previous studies have found that patients with cannabis use disorder, while younger and less comorbid, had higher risk for breakthrough infections of COVID-19 despite vaccination. Wang et al. posit that behavioral factors or adverse pulmonary and immunologic effects of cannabis may contribute to this breakthrough risk (Wang et al. 2021a). Another recent study found that COVID-19 patients with substance use orders have worse outcomes compared to general COVID-19 patients, including increased hospitalization and death (Wang et al. 2021b). This study, however, grouped several substances, including opioids and alcohol use and is not specific to cannabis use.

To our knowledge, this study is one of the first evaluations of the effect of cannabis use on outcomes in patients hospitalized with COVID-19. While previous data have determined the detrimental relationship of tobacco smoking with COVID-19, this study suggests that cannabis may actually lead to reduced disease severity and better outcomes despite a five-fold greater concomitant use of tobacco amongst cannabis users compared to non-users in our study population.

### Limitations

Inaccurate or incomplete documentation in the medical record may bias our findings, as we were beholden to the data contained within the clinical chart, which this retrospective analysis was based on. While the majority of data abstraction was blinded to our study purpose, our authors did evaluate each chart of reported cannabis users to ensure current use. Additionally, cannabis use is self-reported without specific focus on it during routine admission data collection. Thus, we may not have captured all current cannabis users, potentially introducing further selection bias. Additionally, we do not have complete data on the route of cannabis use, frequency or duration, and we were therefore unable to comment on dose response or durability of the potential effects of cannabis consumption. Cannabis users were defined after both automatic and manual data processing and both populations were very well characterized. Given legalized recreational cannabis use in California, our retrospective analysis is less prone to the selection bias and underreporting of cannabis use in comparison to other centers where its use is illegal. Because our focus was also on cannabis use, we were also unable to ascertain alcohol use history and usage of other substances. Therefore, we were unable to factor in substance use disorder into our inverse probability weighting process.

## Conclusions

In this retrospective review of 1831 COVID-19 patients requiring hospital admission, current cannabis use was associated with decreased disease severity. This was demonstrated in lower NIH severity scores as well as less need for oxygen supplementation, ICU admission and mechanical ventilation. While there was a trend toward improved survival in cannabis users, this was not statistically significant. To our knowledge, this is the first study looking at clinical outcomes of cannabis users hospitalized with COVID-19. Further studies, including prospective analyses, will help to better understand the relationship between cannabis and COVID-19 outcomes.

## Supplementary Information

**Additional file 1: Supplemental Table 1**. NIH Severity Score and Definitions (Beigel et al. 2020). **Supplemental Table 2**. Covariate balance after propensity weighting or matching.

## Data Availability

The datasets used and/or analyzed during the current study are available from the corresponding author on reasonable request.

## Abbreviations

ACE2 receptor: Angiotensin converting enzyme 2 receptor
BMI: Body mass index
CBD: Cannabidiol
CDC: Center for Disease Control
COVID-19: Coronavirus Disease 2019
CRP: C-reactive protein
ICD-10 codes: International Classification of Diseases, Tenth Revision
ICU: Intensive care unit
IL-1B: Interleukin 1B
IL-8: Interleukin 8
IRB: Institutional Review Board
NIH: National Institute of Health
OR: Odds Ratio
RedCAP: Research Electronic Data Capture
SARS-CoV-2: Severe acute respiratory syndrome coronavirus 2
sTNFR: Soluble tumor necrosis factor receptors
UCLA: University of California, Los Angeles
WHO: World Health Organization

## References

[CR1] 2020 National Survey of Drug Use and Health (NSDUH) Releases | CBHSQ Data. https://www.samhsa.gov/data/release/2020-national-survey-drug-use-and-health-nsduh-releases. Accessed 1 Oct 2021.

[CR2] Beigel J (2020). Remedesivir for the Treatment of COVID-19 - Final Report. Crit Care.

[CR3] Biotechs, S. Stero Biotechs supports an Exploratory Study of CBD-based treatment for COVID-19 severe patients. https://www.prnewswire.com/il/news-releases/stero-biotechs-supports-an-exploratory-study-of-cbd-based-treatment-for-covid-19-severe-patients-301200798.html. Accessed 1 Oct 2021.

[CR4] Borgonhi EM, Volpatto VL, Ornell F, Rabelo-da-Ponte FD, Kessler FHP (2021). Multiple clinical risks for cannabis users during the COVID-19 pandemic. Addict Sci Clin Pract.

[CR5] Chadi N, Minato C, Stanwick R. Cannabis vaping: Understanding the health risks of a rapidly emerging trend. Paediatr Child Health. 2020;25(Suppl 1):S16–S20. 10.1093/pch/pxaa016 PMC7757764 33390752

[CR6] Colmenero J (2021). Epidemiological pattern, incidence, and outcomes of COVID-19 in liver transplant patients. J Hepatol.

[CR7] Coronavirus Disease (COVID-19) Situation Reports. https://www.who.int/emergencies/diseases/novel-coronavirus-2019/situation-reports. Aaccessed 1 Oct 2021.

[CR8] Dai H, Richter KP (2019). A National Survey of Marijuana Use Among US Adults With Medical Conditions, 2016-2017. JAMA Netw Open.

[CR9] Gold JAW (2021). COVID-19 Case Surveillance: Trends in Person-Level Case Data Completeness, United States, April 5-September 30, 2020. Public Health Rep.

[CR10] Guillon A, Hiemstra PS, Si-Tahar M. Pulmonary immune responses against SARS-CoV-2 infection: harmful or not? Intensive Care Med. 2020;1–4. 10.1007/s00134-020-06170-8. 10.1007/s00134-020-06170-8 PMC7366461 32681297

[CR11] Han S, Mallampalli RK (2015). The acute respiratory distress syndrome: from mechanism to translation. J Immunol.

[CR12] Harris PA (2009). Research electronic data capture (REDCap)--a metadata-driven methodology and workflow process for providing translational research informatics support. J Biomed Inform.

[CR13] Harris PA (2019). The REDCap Consortium: Building an International Community of Software Platform Partners. J Biomed Inform.

[CR14] Hospitalized Adults: Therapeutic Management. COVID-19 Treatment Guidelines https://www.covid19treatmentguidelines.nih.gov/management/clinical-management/hospitalized-adults%2D%2Dtherapeutic-management/. Accessed 1 Oct 2021.

[CR15] Ioannidis JPA (2020). Global perspective of COVID-19 epidemiology for a full-cycle pandemic. Eur J Clin Invest.

[CR16] Maresz K (2007). Direct suppression of CNS autoimmune inflammation via the cannabinoid receptor CB1 on neurons and CB2 on autoreactive T cells. Nat Med.

[CR17] McGilveray IJ (2005). Pharmacokinetics of cannabinoids. Pain Res Manag.

[CR18] Mohammed A, et al. Δ9-Tetrahydrocannabinol Prevents Mortality from Acute Respiratory Distress Syndrome through the Induction of Apoptosis in Immune Cells, Leading to Cytokine Storm Suppression. Int J Mol Sci. 21(17):6244. 10.3390/ijms21176244 PMC7503745 32872332

[CR19] Moore BA, Augustson EM, Moser RP, Budney AJ (2005). Respiratory Effects of Marijuana and Tobacco Use in a U.S. Sample. J Gen Intern Med.

[CR20] Onaivi ES, Sharma V. Cannabis for COVID-19: can cannabinoids quell the cytokine storm? Fut Sci OA. 2020;6:FSO625. 10.2144/fsoa-2020-0124 PMC7451410 32974048

[CR21] Paland N (2021). The Immunopathology of COVID-19 and the Cannabis Paradigm. Front Immunol.

[CR22] Patanavanich R, Glantz SA (2020). Smoking Is Associated With COVID-19 Progression: A Meta-analysis. Nicotine Tob Res.

[CR23] Pink I, et al. C-reactive protein and procalcitonin for antimicrobial stewardship in COVID-19. Infection. 2021. 10.1007/s15010-021-01615-8. 10.1007/s15010-021-01615-8 PMC8140571 34021897

[CR24] Recent Trends in Marijuana Use In Los Angeles County. (2018).

[CR25] Russell C, Rueda S, Room R, Tyndall M, Fischer B (2018). Routes of administration for cannabis use - basic prevalence and related health outcomes: A scoping review and synthesis. Int J Drug Policy.

[CR26] Saadatian-Elahi M, et al. Tobacco smoking and severity of COVID-19: Experience from a hospital-based prospective cohort study in Lyon, France. J Med Virol. 2021:10.1002/jmv.27233. 10.1002/jmv.27233. 10.1002/jmv.27233 PMC8426692 34314045

[CR27] Tashkin DP, Roth MD (2019). Pulmonary effects of inhaled cannabis smoke. Am J Drug Alcohol Abuse.

[CR28] The Lancet Respiratory Medicine (2020). The EVALI outbreak and vaping in the COVID-19 era. Lancet Respir Med.

[CR29] van Breemen RB, et al. Cannabinoids Block Cellular Entry of SARS-CoV-2 and the Emerging Variants. J Nat Prod. 2022. 10.1021/acs.jnatprod.1c00946. 10.1021/acs.jnatprod.1c00946 PMC8768006 35007072

[CR30] Wang L, Wang Q, Davis PB, Volkow ND, Xu R. Increased risk for COVID-19 breakthrough infection in fully vaccinated patients with substance use disorders in the United States between December 2020 and August 2021. World Psychiatry. 2021a:10.1002/wps.20921. 10.1002/wps.20921. 10.1002/wps.20921 PMC8661963 34612005

[CR31] Wang QQ, Kaelber DC, Xu R, Volkow ND (2021). COVID-19 risk and outcomes in patients with substance use disorders: analyses from electronic health records in the United States. Mol Psychiatry.

