# Supplementary material for: Cannabis consumption is associated with lower COVID-19 severity among hospitalized patients: a retrospective cohort analysis
Source: J Cannabis Res. 2022 Aug 5;4:46. doi: 10.1186/s42238-022-00152-x (PMC9356466; doi:10.1186/s42238-022-00152-x)
Supplement: Supplementary file 1 — Additional file 1: Supplemental Table 1. NIH Severity Score and Definitions (Beigel et al. 2020). Supplemental Table 2. Covariate balance after propensity weighting or matching. [file 42238_2022_152_MOESM1_ESM.docx]

*Supplemental Table 1: NIH Severity Score and Definitions^21^*

| **Scale** | **Definition** | |
| --- | --- | --- |
| 1 | Not hospitalized and no limitations of activities | |
| 2 | Not hospitalized, with limitation of activities, home oxygen requirement, or both | |
| 3 | Hospitalized, not requiring supplemental oxygen and no longer requiring ongoing medical care | |
| 4** | Hospitalized, not requiring supplemental oxygen but requiring ongoing medical care | |
| 5 | Hospitalized, requiring any supplemental oxygen | |
| 6 | Hospitalized, requiring noninvasive ventilation or use of high-flow oxygen devices | |
| 7 | Hospitalized, receiving invasive mechanical ventilation or extracorporeal membrane oxygenation (ECMO) | |
| 8 | Death | |
| **Minimum score for analyzed cohort | |  |

*Supplemental Table 2: Covariate balance after propensity weighting or matching*

| **Covariates** | **Standardized  Raw  Difference** | **^1^Standardized Weighted Difference** | **^2^Standardized Matched Difference** |
| --- | --- | --- | --- |
| Age | -0.97 | -0.08 | -0.02 |
| Age^2^ | -0.93 | -0.08 | -0.01 |
| BMI (kg/m^2^ | -0.08 | -0.11 | -0.19 |
| BMI^2^ (kg/m^2^) | -0.05 | -0.12 | -0.16 |
| Male Sex | 0.17 | 0.16 | 0.15 |
| Black | 0.17 | -0.14 | -0.13 |
| Latinx | -0.26 | -0.07 | -0.20 |
| Hispanic-White | -0.11 | -0.02 | 0.24 |
| Asian/Pacific Islander | -0.14 | 0.05 | 0.01 |
| Unknown/Multiracial | -0.15 | 0.01 | -0.05 |
| Former Smoker | -0.04 | 0.03 | 0.09 |
| Current Smoker | 0.50 | 0.00 | 0.01 |
| Diabetes mellitus | -0.30 | 0.09 | 0.13 |
| Cardiac Disease | -0.21 | -0.09 | 0.03 |
| Chronic Kidney Disease | -0.24 | -0.05 | 0.09 |
| Chronic Pulmonary Disease | -0.01 | -0.29 | -0.26 |
| Chronic Liver Disease | -0.14 | -0.17 | -0.16 |
| Systemic Steroid Use | -0.41 | -0.02 | -0.06 |
| Antibacterial Use | -0.35 | -0.03 | 0.10 |
| ^1^Inverse-Probability-Weighted Regression Adjustment; ^2^Propensity-Score Matching | | | |
